# The Urban-Rural Gradient In Asthma: A Population-Based Study in Northern Europe

**DOI:** 10.3390/ijerph13010093

**Published:** 2015-12-30

**Authors:** Signe Timm, Morten Frydenberg, Christer Janson, Brittany Campbell, Bertil Forsberg, Thorarinn Gislason, Mathias Holm, Rain Jogi, Ernst Omenaas, Torben Sigsgaard, Cecilie Svanes, Vivi Schlünssen

**Affiliations:** 1 Department of Public Health, Danish Ramazzini Centre, Aarhus University, DK-8000 Aarhus C, Denmark; morten@ph.au.dk (M.F.); ts@ph.au.dk (T.S.); vs@ph.au.dk (V.S.); 2 Department of Medical Sciences: Respiratory, Allergy and Sleep Research, Uppsala University, 751 05 Uppsala, Sweden; christer.janson@medsci.uu.se; 3 Allergy and Lung Health Unit, Centre for Epidemiology and Biostatistics, Melbourne School of Population and Global Health, The University of Melbourne, Victoria 3010, Australia; brittany.campbell@unimelb.edu.au; 4 Division of Occupational and Environmental Medicine, Department of Public Health and Clinical Medicine, Umeå University, 901 87 Umeå, Sweden; bertil.forsberg@envmed.umu.se; 5 Department of Respiratory Medicine and Sleep, Landspitali University Hospital, Reykjavik IS-108, Iceland; thorarig@landspitali.is; 6 Faculty of Medicine, University of Iceland, Reykjavik IS-101, Iceland; 7 Department of Occupational and Environmental Medicine, Sahlgrenska University Hospital, Gothenburg 405 30, Sweden; mathias.holm@amm.gu.se; 8 Lung Clinic, Tartu University Hospital, Tartu 50406, Estonia; Rain.Jogi@kliinikum.ee; 9 Institute of Clinical Science, University of Bergen, 5020 Bergen, Norway; ernst.omenaas@helse-bergen.no (E.O.); cecilie.svanes@helse-bergen.no (C.S.); 10 National Research Center for Working Environment, Copenhagen DK-2100, Denmark

**Keywords:** asthma, early life environment, farming, microbial exposure, urban-rural gradient, hygiene hypothesis, RHINE

## Abstract

The early life environment appears to have a persistent impact on asthma risk. We hypothesize that environmental factors related to rural life mediate lower asthma prevalence in rural populations, and aimed to investigate an urban-rural gradient, assessed by place of upbringing, for asthma. The population-based Respiratory Health In Northern Europe (RHINE) study includes subjects from Denmark, Norway, Sweden, Iceland and Estonia born 1945–1973. The present analysis encompasses questionnaire data on 11,123 RHINE subjects. Six categories of place of upbringing were defined: farm with livestock, farm without livestock, village in rural area, small town, city suburb and inner city. The association of place of upbringing with asthma onset was analysed with Cox regression adjusted for relevant confounders. Subjects growing up on livestock farms had less asthma (8%) than subjects growing up in inner cities (11%) (hazard ratio 0.72 95% CI 0.57–0.91), and a significant urban-rural gradient was observed across six urbanisation levels (*p* = 0.02). An urban-rural gradient was only evident among women, smokers and for late-onset asthma. Analyses on wheeze and place of upbringing revealed similar results. In conclusion, this study suggests a protective effect of livestock farm upbringing on asthma development and an urban-rural gradient in a Northern European population.

## 1. Introduction

Knowledge of the aetiology of asthma remains poor, although asthma prevalence has risen steeply in recent years. This rise has been ascribed to the 20th century environmental changes, including extensive urbanisation. Growing up in cities is reported to be associated with higher asthma risk than growing up on farms; this observation has contributed to the hypothesis that limited exposure to microbial diversity plays a key role in asthma aetiology [1,2]. Supporting this hypothesis, Ege *et al.* found a wider range of microbial species in children growing up on a farm, and several studies suggests that endotoxin levels in the indoor environment varies according to the level of urbanisation [3,4,5]. Moreover, living in rural areas in close distance to neighbouring farms seems to lower the asthma risk too [6]. Protective effects on asthma seems to be limited to early life exposure, since occupational farm exposure later in life may cause increased risk of respiratory symptoms and also asthma [7]. However, recent evidence of the effect of farm upbringing on asthma is inconsistent [8,9,10,11,12,13]. Growing up on a farm may reflect factors in a farm environment, however, it may also reflect not growing up in an urban environment with presence of several risk factors for asthma such as smoking and air pollution [14]. Current evidence tends to focus on the effect of farm upbringing compared with city or non-farm upbringing, and it remains unclear whether an urban-rural gradient exists for asthma as suggested for IgE mediated sensitisation and inflammatory bowel diseases [15,16]. Investigating the urban-rural gradient in asthma will push our knowledge a step further by revealing if the relative size of the urban-rural environments also is a decisive factor. If observed, an urban-rural gradient may indicate a causal relationship and raise evidence for a dose-response like relationship between microbial diversity and development of asthma. This will provide a more detailed understanding of the early life origin of asthma. The aim of this study was to investigate the urban-rural gradient in place of upbringing for asthma in a population-based multi-centre study in Northern Europe.

## 2. Material and Methods

### 2.1. Study Population

This study is based on data from a subpopulation of The European Community Respiratory Health Survey (ECRHS). The original study population included >150,000 randomly selected men and women born between 1945 and 1973, who participated in ECRHS stage 1 during 1989–1992. Each of the 48 participating centres recruited at least 1500 men and 1500 women aged 20–44 years [17]. The Respiratory Health in Northern Europe (RHINE) study followed up on 21,659 subjects from the seven study centres located in Northern Europe—Reykjavik in Iceland; Bergen in Norway; Umeå, Uppsala and Gothenburg in Sweden; Tartu in Estonia; and Aarhus in Denmark. In 1999–2001, all RHINE subjects were sent a postal questionnaire (RHINE II), which was answered by 16,106 subjects (74%). At follow-up in 2010–2012, altogether 13,499 subjects (62%) responded to the RHINE III questionnaire [18]. The present analysis is based on this follow-up. The local Science Ethics Committees approved the study for each study centre, and informed consent was obtained from all study participants.

### 2.2. Questionnaire Information

All information was obtained from the RHINE III questionnaire in 2010–2012. Information was obtained from a standardised postal questionnaire. A formal forward/backward translation of the questionnaires was performed to ensure validity and homogeneity between study centres.

*Asthma* was obtained in RHINE III and defined as an affirmative answer to either “Do you have or have you ever had asthma?” or “Have you ever had asthma diagnosed by a doctor?” and a retrospectively reported age of onset. Allergic asthma was defined as an affirmative answer to both hay fever and asthma. *Wheeze* was defined as an affirmative answer to “Have you ever had wheezing or whistling in your chest?” and a retrospectively reported age of onset.

*Place of upbringing* was obtained in RHINE III and defined as the place the subject lived most of the time under the age of five years with response categories (1) farm with livestock; (2) farm without livestock; (3) village in rural area; (4) small town; (5) suburb of city; and (6) inner city.

*Confounding variables* were selected a priori on the basis of a literature search. These were age, sex, centre, smoking, body silhouette at 8 years of age, parental smoking in childhood and parental asthma. Smoking exposures were categorised as current, ex- and never-smokers. Anthropometric characteristics were measured by recalled body silhouette at age 8 years classified as lean, normal and obese. Parental smoking was defined by regular smoking by either parent during childhood, and parental asthma was defined by either biological parent ever suffering from asthma.

### 2.3. Statistical Analysis

Eligible subjects had information on the basic variables place of upbringing, asthma/wheeze and time of onset. However, the final analyses were conducted on subjects with complete information on all variables included in the models (hereafter called the study population) to ensure a constant number throughout all analyses. Data were analysed by Cox regression models with age as time scale and presented by hazard ratios (HR) with corresponding 95% confidence intervals (95% CI). The proportional hazard assumption was tested by log-log plots and found acceptable. Although the data collection in this study is cross sectional, the information given by the subjects specifies the exact time of exposure and outcome, which warranting the data to be analysed as longitudinal data. Subjects were assumed to be at risk from birth and censored at the time of asthma/wheeze onset, respectively, or at the end of follow up, whichever appeared first. HRs were presented for each urbanisation level respectively, and furthermore HRs comparing two adjacent urbanisation levels were presented including p values for an urban-rural trend. Data were analysed as crude, adjusted for non-time dependent covariates (age, sex, centre and parental asthma) and further adjusted for time-dependent covariates (smoking, body silhouette at 8 years of age and parental smoking in childhood). Smoking was analysed as a time-dependent covariate. Adjustment for centre was done by stratifying. It takes large strength to get significant interactions, and as there are differences between centres with regard to farming environment, language, translations *etc.* it was therefore, a priori, determined to stratify by centre, which is usually required for all the analyses of this multi-centre study, whether interaction by centre for the particular associations investigated in this paper would be significant or not.

Analyses were conducted separately for the two outcomes, asthma and wheeze. Additional analyses included estimation of prevalence and incidence (with time at risk starting from birth), and Cox regression stratified by sex, time of onset, smoking status, centre and allergy status, respectively. As there is no strong consensus in the literature about the cut-off point for early-onset *versus* late-onset asthma, the cut-off was set at 10 years of age in line with previous studies in the ECRHS/RHINE cohort, to ensure that asthma in puberty was not defined as early-onset (childhood) asthma. Interaction was tested by adding the interaction to the model and testing it by Wald test. A sensitivity analysis on incident cases in the follow-up period 1989–2010 was performed to explore the impact of selection bias. Statistics were calculated using STATA 12.1 (STATA Corp., College Station, TX, USA) and all *p*-values were two-sided with significance level 5%.

## 3. Results

Of the 13,499 RHINE III responders, data on exposure and outcomes were available for 12,441 eligible subjects. Basic characteristics of the eligible subjects and the study population (*N* = 11,123) are shown in Table 1 and characteristics according to centre are given as online supplementary (Table S1, Supplementary Material). The study population included 1181 cases with asthma (10.6%) and 2133 cases with wheeze (19.1%). This corresponds to an incidence of 2.14 (95% CI 2.02–2.27) per 1000 person-years for asthma and 3.94 (95% CI 3.78–4.11) per 1000 person-years for wheeze. Subjects in the six exposure groups were comparable regarding age, sex, parental asthma and body silhouettes at 8 years.

**Table 1:** Characteristics of the study population and the eligible subjects.

	Inner City	Suburb of City	Small Town	Village in Rural Area	Farm without Livestock	Farm with Livestock	Study Population	Eligible Subjects *
Subjects, N	1725	3337	2720	1599	250	1492	11,123	12,441
Age in 2011, mean ± SD	53.5 ± 7.1	52.0 ± 7.1	52.2 ± 7.0	54.14 ± 7.1	52.5 ± 6.7	55.6 ± 6.6	53.1 ± 7.1	53.0 ± 7.1
Sex, N (%F)	872 (51%)	1765 (53%)	1441 (53%)	915 (57%)	126 (50%)	850 (57%)	5969 (54%)	6612 (53%)
Smoking status								
Current smoker, N (%)	494 (29%)	824 (24%)	581 (21%)	311 (20%)	64 (26%)	328 (22%)	2602 (23%)	2757 (22%)
Ex-smoker, N (%)	409 (34%)	822 (25%)	647 (24%)	428 (26%)	60 (24%)	348 (23%)	2714 (24%)	2842 (23%)
Never smokers, N (%)	670 (39%)	1378 (41%)	1281 (47%)	744 (47%)	108 (43%)	702 (47%)	4883 (44%)	5061 (41%)
Age at smoke start, mean ± SD	16.9 ± 4.4	17.2 ± 4.3	17.4 ± 4.3	17.0 ± 4.1	17.3 ± 4.3	17.9 ± 4.9	17.3 ± 4.4	17.3 ± 4.4
Parental smoking:								
No parents smoke, N (%)	430 (25%)	976 (29%)	875 (32%)	538 (34%)	74 (30%)	641 (43%)	3534 (32%)	3976 (32%)
One parent smoke, N (%)	630 (37%)	1284 (39%)	942 (35%)	623 (39%)	95 (38%)	597 (40%)	4171 (38%)	4637 (37%)
Both parents smoke, N (%)	593 (34%)	972 (29%)	815 (30%)	373 (23%)	73 (29%)	193 (13%)	3019 (27%)	3343 (27%)
Don’t know, N (%)	72 (4%)	105 (3%)	88 (3%)	65 (4%)	8 (3%)	61 (4%)	399 (4%)	462 (4%)
Body silhouette at 8y								
1–3 (lean), N (%)	1422 (82%)	2742 (82%)	2203 (81%)	1312 (82%)	211 (84%)	1194 (80%)	9084 (82%)	9743 (78%)
4–6 (normal), N (%)	281 (16%)	552 (17%)	479 (18%)	269 (17%)	37 (15%)	269 (18%)	1887 (17%)	1996 (16%)
7–9 (obese), N (%)	22 (1%)	43 (1%)	38 (1%)	18 (1%)	2 (1%)	29 (2%)	152 (1%)	159 (1%)
Centre								
Aarhus (DK), N (%)	351 (20%)	600 (18%)	475 (17%)	271 (17%)	26 (11%)	229 (15%)	1952 (18%)	2182 (18%)
Reykjavik (IS), N (%)	297 (17%)	664 (20%)	454 (17%)	68 (4%)	20 (8%)	131 (9%)	1634 (15%)	1862 (15%)
Bergen (NO), N (%)	343 (20%)	580 (17%)	488 (18%)	79 (5%)	131 (52%)	231 (15%)	1852 (17%)	2050 (16%)
Gothenburg (SE), N (%)	256 (15%)	660 (20%)	235 (9%)	185 (12%)	15 (6%)	95 (6%)	1446 (13%)	1631 (13%)
Umeaa (SE), N (%)	94 (5%)	137 (4%)	464 (17%)	499 (31%)	28 (11%)	432 (29%)	1654 (15%)	1840 (15%)
Uppsala (SE), N (%)	256 (15%)	380 (11%)	462 (17%)	375 (23%)	24 (10%)	192 (13%)	1689 (15%)	1859 (15%)
Tartu (EE), N (%)	128 (7%)	316 (9%)	142 (5%)	122 (8%)	6 (2%)	182 (12%)	896 (8%)	1017 (8%)
Parental asthma								
Mother, N (%)	144 (8%)	280 (8%)	208 (8%)	120 (8%)	23 (9%)	123 (8%)	898 (8%)	1021 (8%)
Father, N (%)	80 (5%)	162 (5%)	125 (5%)	87 (5%)	17 (7%)	78 (5%)	549 (5%)	615 (5%)
No parental asthma, N (%)	1492 (86%)	2873 (86%)	2379 (87%)	1378 (86)	208 (83%)	1282 (86%)	9612 (86%)	9292 (75%)
Both parents asthma, N (%)	9 (1%)	22 (1%)	8 (1%)	14 (1%)	2 (1%)	9 (1%)	64 (1%)	70 (1%)
Hay fever								
Yes, N (%)	466 (27%)	805 (24%)	706 (26%)	364 (23%)	55 (22%)	301 (20%)	2697 (24%)	2998 (24%)

* = Numbers may vary due to missing data.

**Figure 1:**
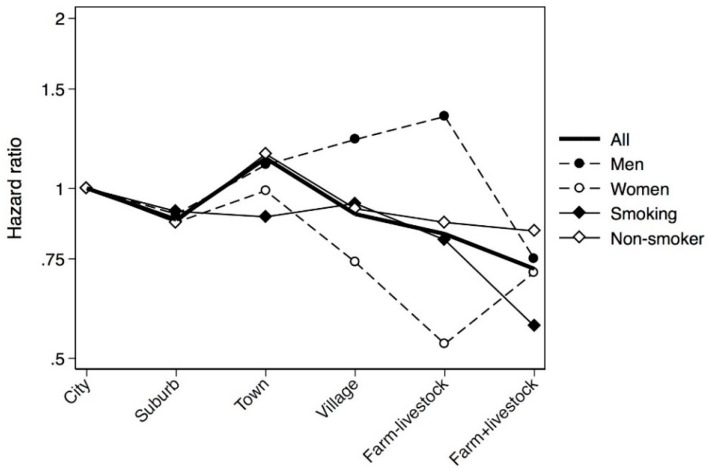
Cox regression subgroup analyses on asthma presented as HR adjusted for age, sex, centre, parental asthma, smoking, body silhouette at 8 years of age and parental smoking in childhood.

**Figure 2:**
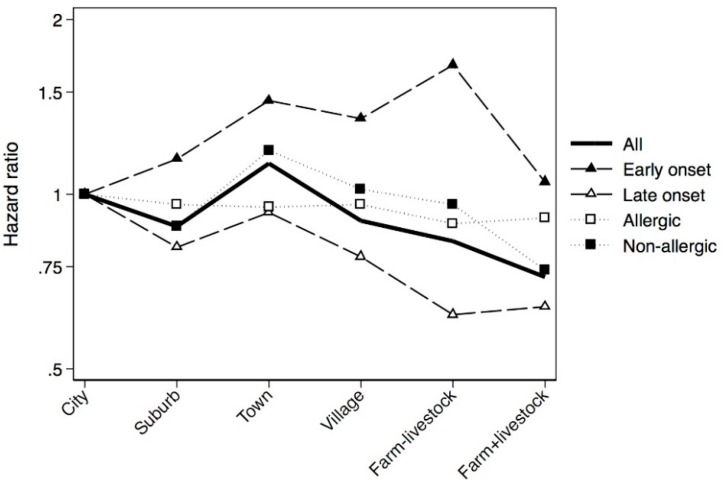
Cox regression analyses on asthma phenotypes presented as HR adjusted for age, sex, centre, parental asthma, smoking, body silhouette at 8 years of age and parental smoking in childhood.

Subjects who grew up in a city were more likely to be current smokers and exposed to parental smoking in childhood. Growing up on a farm without livestock was more common among Norwegians than other participating centres. In Cox regression analyses, subjects who grew up on a livestock farm were significantly less likely to suffer from asthma than subjects who grew up in a city, and a significant urban-rural gradient was observed across the six urbanisation levels (Table 2). The incidence rates according to place of upbringing varied from 1.59 (95% CI 1.34–1.90) for living on farms with livestock to 2.55 (95% CI 2.29–2.84) for living in small towns (Table 2). Sub-analyses revealed that farm upbringing was protective only among smokers, and an urban-rural gradient was also only present among smokers (Table 3). The sex-specific analyses showed that the effect of livestock farming was similar among men and women, however, upbringing in rural areas and on farm without livestock was only protective in women. An urban-rural gradient was only detected among women (Table 3). Furthermore, livestock farming was protective only against late-onset asthma, and an urban-rural gradient was only present for this phenotype (Table 4). Adjusted urban-rural gradients for the certain subgroups and phenotypes are shown in Figure 1 and Figure 2.

**Table 2:** Descriptive data on asthma and wheeze and Cox regression analyses on asthma for all presented as HR (95% CI).

	Inner City	Suburb of City	Small Town	Village in Rural Area	Farm without Livestock	Farm with Livestock	HR for Urban-Rural Trend “	*p* for Urban-Rural Trend
Cases with asthma N (%)	194 (11%)	334 (10%)	334 (12%)	167 (10%)	27 (11%)	125 (8%)		
Incidence of asthma per 1000 pyr (95% CI)	2.25 (1.95–2.59)	2.06 (1.85–2.29)	2.55 (2.29–2.84)	2.07 (1.78–2.41)	2.22 (1.52–3.24)	1.59 (1.34–1.90)		
Mean age of asthma onset ± SD	26.6 ± 15.8	24.5 ± 15.6	23.6 ± 16.4	24.9 ± 17.6	22.1 ± 17.3	27.3 ± 17.9		
Cases with wheeze N (%)	368 (21%)	628 (19%)	541 (20%)	288 (18%)	47 (19%)	261 (17%)		
All								
Crude	1	0.91 (0.76–1.09)	1.13 (0.95–1.35)	0.92 (0.75–1.13)	0.98 (0.66–1.47)	0.71 (0.57–0.89)	0.95 (0.92–0.99)	0.01
Adjusted 1 °	1	0.87 (0.73–1.04)	1.03 (0.86–1.23)	0.90 (0.72–1.11)	0.83 (0.55–1.24)	0.71 (0.56–0.89)	0.95 (0.91–0.99)	0.01
Adjusted 2 *	1	0.88 (0.73–1.05)	1.04 (0.87–1.24)	0.90 (0.73–1.12)	0.83 (0.56–1.25)	0.72 (0.57–0.91)	0.95 (0.92–0.99)	0.02

° = Adjusted for sex, age, centre and parental asthma; * = Adjusted for sex, age, centre, parental asthma, smoking, bodyshape at 8 years and parental smoking in childhood; “ = comparing two adjacent levels of urbanisation.

**Table 3:** Cox regression analyses on asthma stratified by sex and smoking status presented as HR (95% CI).

	Inner City	Suburb of City	Small Town	Village in Rural Area	Farm without Livestock	Farm with Livestock	HR for Urban-Rural Trend “	*p* for Urban-Rural Trend
Men								
Crude	1	0.90 (0.68–1.20)	1.20 (0.91–1.59)	1.17 (0.85–1.61)	1.60 (0.95–2.70)	0.70 (0.48–1.02)	0.98 (0.93–1.04)	0.62
Adjusted 1 °	1	0.90 (0.67–1.19)	1.11 (0.83–1.47)	1.22 (0.88–1.71)	1.34 (0.79–2.28)	0.75 (0.51–1.10)	0.99 (0.93–1.06)	0.77
Adjusted 2 *	1	0.90 (0.67–1.19)	1.10 (0.83–1.46)	1.22 (0.87–1.71)	1.34 (0.79–2.28)	0.75 (0.51–1.11)	0.99 (0.93–1.06)	0.81
Women								
Crude	1	0.90 (0.72–1.13)	1.07 (0.85–1.34)	0.76 (0.58–0.99)	0.59 (0.31–1.12)	0.69 (0.52–0.91)	0.93 (0.88–0.97)	<0.01
Adjusted 1 °	1	0.86 (0.69–1.08)	0.97 (0.77–1.22)	0.73 (0.55–0.97)	0.52 (0.27–0.99)	0.68 (0.51–0.91)	0.93 (0.88–0.97)	<0.01
Adjusted 2 *	1	0.87 (0.70–1.10)	0.99 (0.79–1.25)	0.74 (0.56–0.98)	0.53 (0.27–1.00)	0.71 (0.53–0.94)	0.93 (0.88–0.98)	<0.01
Smoking								
Crude	1	0.96 (0.72–1.26)	0.93 (0.70–1.25)	0.90 (0.65–1.26)	0.96 (0.50–1.86)	0.59 (0.40–0.87)	0.92 (0.86–0.98)	0.01
Adjusted 1 °	1	0.90 (0.69–1.19)	0.88 (0.66–1.19)	0.94 (0.66–1.33)	0.80 (0.41–1.56)	0.58 (0.39–0.86)	0.92 (0.86–0.98)	0.02
Adjusted 2 *	1	0.91 (0.69–1.19)	0.89 (0.66–1.19)	0.94 (0.66–1.33)	0.81 (0.42–1.58)	0.57 (0.38–0.85)	0.92 (0.86–0.98)	0.01
Not smoking								
Crude	1	0.89 (0.70–1.12)	1.26 (1.01–1.57)	0.95 (0.73–1.23)	1.01 (0.61–1.68)	0.80 (0.60–1.06)	0.98 (0.93–1.02)	0.28
Adjusted 1 °	1	0.87 (0.69–1.09)	1.14 (0.91–1.43)	0.91 (0.69–1.20)	0.85 (0.51–1.43)	0.82 (0.61–1.10)	0.97 (0.93–1.02)	0.29
Adjusted 2 *	1	0.87 (0.69–1.10)	1.15 (0.92–1.45)	0.92 (0.70–1.21)	0.87 (0.51–1.45)	0.84 (0.63–1.12)	0.98 (0.93–1.03)	0.39

° = Adjusted for sex, age, centre and parental asthma; * = Adjusted for sex, age, centre, parental asthma, smoking, bodyshape at 8 years and parental smoking in childhood; “ = comparing two adjacent levels of urbanisation.

**Table 4:** Cox regression analyses on asthma phenotypes presented as HR (95% CI). Allergic asthma was defined as presence of both hay fever and asthma.

	Inner City	Suburb of City	Small Town	Village in Rural Area	Farm without Livestock	Farm with Livestock	HR for Urban-Rural Trend “	*p* for Urban-Rural Trend
Early onset (≤10 years of age)								
Crude	1	1.14 (0.78–1.65)	1.56 (1.08–2.25)	1.43 (0.95–2.16)	1.91 (0.98–3.73)	0.98 (0.62–1.55)	1.02 (0.95–1.09)	0.58
Adjusted 1 °	1	1.15 (0.79–1.68)	1.44 (1.00–2.09)	1.34 (0.88–2.04)	1.65 (0.84–3.23)	1.03 (0.65–1.64)	1.02 (0.95–1.10)	0.61
Adjusted 2 *	1	1.15 (0.79–1.68)	1.45 (1.00–2.10)	1.35 (0.89–2.06)	1.67 (0.85–3.27)	1.05 (0.65–1.68)	1.02 (0.95–1.11)	0.54
Late onset (>10 years of age)								
Crude	1	0.85 (0.70–1.04)	1.02 (0.83–1.24)	0.79 (0.62–1.00)	0.74 (0.44–1.24)	0.64 (0.50–0.83)	0.93 (0.89–0.97)	<0.01
Adjusted 1 °	1	0.80 (0.66–0.98)	0.92 (0.75–1.13)	0.78 (0.60–1.00)	0.61 (0.36–1.02)	0.63 (0.48–0.82)	0.92 (0.88–0.97)	<0.01
Adjusted 2 *	1	0.81 (0.66–0.99)	0.93 (0.76–1.15)	0.78 (0.61–1.01)	0.62 (0.37–1.04)	0.64 (0.49–0.83)	0.93 (0.88–0.97)	<0.01
Allergic asthma								
Crude	1	0.96 (0.76–1.22)	1.06 (0.83–1.35)	1.10 (0.83–1.45)	1.00 (0.55–1.81)	0.95 (0.70–1.29)	1.00 (0.95–1.06)	0.90
Adjusted 1 °	1	0.96 (0.75–1.22)	0.95 (0.74–1.21)	0.96 (0.72–1.28)	0.89 (0.49–1.63)	0.90 (0.66–1.23)	0.98 (0.93–1.04)	0.52
Adjusted 2 *	1	0.96 (0.75–1.22)	0.95 (0.75–1.22)	0.96 (0.72–1.29)	0.89 (0.49–1.63)	0.91 (0.67–1.25)	0.98 (0.93–1.04)	0.59
Non-allergic asthma								
Crude	1	0.91 (0.70–1.20)	1.25 (0.95–1.63)	0.89 (0.65–1.22)	1.13 (0.64–1.99)	0.66 (0.48–0.94)	0.95 (0.89–1.00)	0.05
Adjusted 1 °	1	0.87 (0.66–1.14)	1.18 (0.90–1.55)	1.01 (0.72–1.40)	0.93 (0.52–1.66)	0.72 (0.51–1.02)	0.96 (0.91–1.02)	0.21
Adjusted 2 *	1	0.88 (0.67–1.15)	1.19 (0.91–1.56)	1.02 (0.73–1.42)	0.96 (0.54–1.70)	0.74 (0.52–1.04)	0.97 (0.91–1.03)	0.28

° = Adjusted for sex, age, centre and parental asthma; * = Adjusted for sex, age, centre, parental asthma, smoking, bodyshape at 8 years and parental smoking in childhood; “ = comparing two adjacent levels of urbanisation.

**Figure 3:**
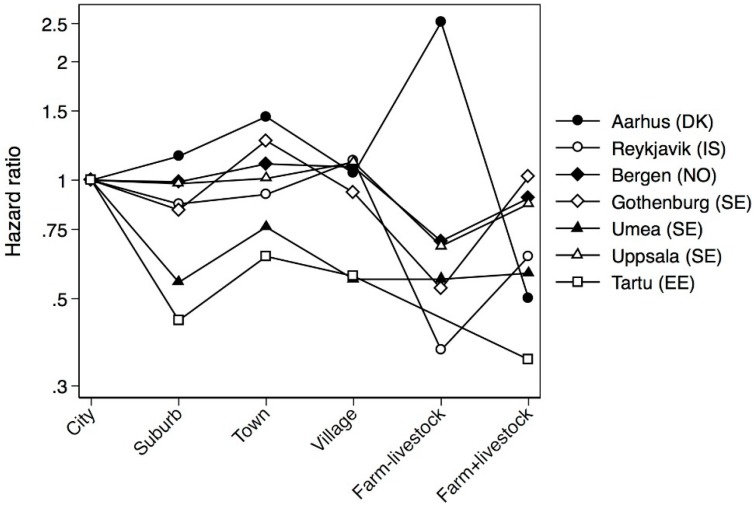
Centre specific Cox regression analyses on asthma presented as HR adjusted for age, sex, centre, parental asthma, smoking, body silhouette at 8 years of age and parental smoking in childhood.

The centre-specific analyses showed geographic variation in the effect of livestock farm upbringing (Figure 3). For Tartu livestock farm upbringing was protective (HR 0.35 95% CI 0.14–0.88), while no association was found for Gothenburg (HR 1.02 95% CI 0.45–2.30). However, there was no significant interaction between place of upbringing and centre (*p* = 0.92), and no urban-rural gradient for the respective centres.

Analyses on wheeze and place of upbringing revealed similar results except for suburb of city, which seems to be protective among non-smokers (HR 0.80 95% CI 0.65–0.98) and allergic subjects (HR 0.78 95% CI 0.64–0.96). Furthermore, the urban-rural gradient for smokers and for late-onset asthma was not confirmed (Table S2 and S3, Supplementary Material).

Both crude and partly adjusted sub-analyses on all eligible subjects (*N* = 12,441) revealed similar results as the fully adjusted analyses. In addition, the sensitivity analysis performed on incident asthma cases during the follow-up period 1989–2010 (487 cases) showed similar results, however the results were not statistically significant. Furthermore, removal of parental asthma from the adjusted model barely changed the results.

## 4. Discussion

In this population-based study, subjects growing up on livestock farms had significantly less asthma than subjects growing up in cities, and an urban-rural gradient in asthma development was observed across six levels of urbanisation. This urban-rural gradient was evident only among women, smokers and only for a late-onset asthma phenotype.

To our knowledge, this is the first study to investigate the urban-rural gradient of asthma applying survival analyses in a population-based study. Although the data collection in this present study is cross-sectional, the given information includes the exact time of exposure and occurrence of outcome, which warrant survival analyses of retrospectively reported onset of asthma with time at risk starting from birth.

As a potential limitation of the present study, information on all variables of interest was self-reported and a potential risk of recall bias and misclassification therefore exists. However, an analysis of the ECRHS showed consistent, long-term repeatability in adults reporting of childhood events, and that the misclassification was not associated with asthma [19]. However, this analyses was for instance not on anthropometric characteristics and we believe that silhouette at 8 years of age might be subject to recall bias. While some misclassification in reporting early life factors is likely, it seems unlikely that such error should be differential with regard to current symptoms although such error cannot be excluded.

Another weakness of the study is the lack of information about where the subjects lived after their first 5 years of life, as this might also have an impact on the risk of developing asthma. Farming exposure can be protective even after 5 years of age as shown by Douwes *et al.* who have found adults to be protected from asthma regardless of the timing of farming exposure. The strongest protection was found in those with current (adult) and childhood exposure, and least in those with only childhood exposure [20]. However, it is likely that most people exposed to farming after the age of 5 years also were exposed before that.

Both self-reported asthma and doctor-diagnosed asthma have a high specificity and a low sensitivity [21]. Asthma prevalence in the present study (10.6%) was comparable to asthma prevalence estimates from other Nordic studies [22,23]. Age of onset may also be subject to misclassification and recall bias. However, a previous analysis of the RHINE population showed approximately 90% reporting the correct year of asthma onset (±1 year) according to their clinical asthma diagnosis [24].

The 1318 subjects excluded from this study due to missing data were primarily excluded due to missing smoking data (812 subjects, among them 89 cases with asthma and 144 cases with wheeze). The prevalence of missing data was equally distributed across the six exposure categories, and we therefore assess the dropout to be non-differential. Furthermore, an analysis of loss to follow-up in the RHINE population suggested that asthma prevalence was somewhat higher among long-term responders, but risk associations were not affected by the non-response [18]. In addition, the sensitivity analysis performed on incident cases during the follow-up period 1989–2010 showed similar results, which supports a minimal impact of selection bias at baseline. However, this analysis will only make inferences about late-onset asthma and did not take into account that asthma-cases may be more likely to participate in the follow-up as well.

A methodological limitation is the retrospective procedures for estimates of risk and occurrence of disease. These estimates require that subjects were alive and reachable for follow-up in 2010–2012, and that they were able to retrospectively report the time of onset for asthma or wheeze. The Cox regression models assume subjects to be at risk during their whole life, even though the marker of microbial exposure is only valid for the first 5 years of life. Recent studies suggest that early life exposures may induce life-long effects on immunoregulatory properties, but it may still be questioned whether this is a reasonable assumption [25]. Because of these conditions the estimates must be interpreted with this limitation in mind.

The findings from this study are overall comparable to current evidence. In line with our findings, Lawson *et al.* found an urban-rural gradient of asthma but not wheeze in a cross-sectional study among adolescents in Canada with only three categories of residence [26].

In a recent review, farm upbringing was found to be protective against asthma and asthma-like symptoms in several studies performed in the three large European cohorts ALEX, PARSIFAL and GABRIELA [2]. However, no association was found between farm upbringing and the risk of asthma or wheeze in the ECRHS study [13]. Different farm locations and farming practices within Europe may explain the heterogeneity in these results. Furthermore, Bråbäck *et al.* observed a cohort effect when investigating trends of asthma through three decades in Sweden, as the protective effect of farm living on asthma was only observed in cohorts born after 1970 [11]. Similarly, a cohort effect of farming was observed for inflammatory bowel disease in the RHINE population, where only subjects born after 1952 gained protection from livestock farm upbringing [15]. Post hoc analyses comparing subjects born before and after 1959 did not confirm a cohort effect of asthma in this study.

The sex-specific effects among subjects growing up on a farm without livestock or in a village in a rural area are in line with findings from the GABRIELA study in Germany, showing the protective effect of growing up on a farm to be slightly more pronounced among girls [27]. The GABRIELA study did not take livestock into account, and we therefore assess the subjects in their “farm” group to be comparable with the subjects from farms with livestock, farms without livestock and villages in rural area in our study. The explanation for these sex specific findings is unknown, however, we can imagine girls growing up in rural areas to be more in contact with “farming exposure” from horses, stables ect. and boys being more exposed to motor exhaust from tractors *etc.* both before and after the age of 5 years, but this is purely speculative. A Danish study on farming students did not find any differences in asthma risk according to sex [7].

The protective effect from livestock farming being strongest among smokers was an unexpected finding, although the interaction was not statistically significant (*p* > 0.1). Smokers with asthma-like symptoms are less likely to receive a diagnosis of asthma than their non-smoking peers, and together with the healthy smoker effect, this will induce bias tending to underestimate asthma among smokers [28,29]. However, it is unlikely that such misclassification is differential with regard to place of upbringing, and we therefore expect the misclassification to be non-differential. A recent Danish study suggests smoking to be a risk factor and farm upbringing to be a protective factor for development of late-onset asthma, but this study did not explore smoking as a potential effect modifier [7].

Smoking is less prevalent in farming areas [26], which is confirmed in this study (Table 1), but adjustment for smoking did not change the results markedly, and is probably not an explanation for the association between farm upbringing and asthma. Furthermore, a study on children shows a protective effect from farm exposure despite the children being exposed to maternal smoking as well [30].

Our results on specific phenotypes suggest a tendency towards a stronger protective effect from livestock farming on late-onset asthma and non-allergic asthma. Comparable to our findings, Omland *et al.* found farm upbringing to be protective against development of late onset asthma among 16–26 year old Danes, but in contrast with our findings, Ege *et al.* also found farm living to be protective against early onset asthma among 5–13 year old European children in a cross-sectional study [7,12]. Adult exposures and particularly occupational factors and air pollution may play a role in our findings and lead to higher risk of late-onset asthma among city-dwellers, hence we believe that place of residence during childhood and adulthood are correlated. In contrast with our findings, Ege *et al.* and Omland *et al.* found farm upbringing to be protective against both allergic and non-allergic asthma [7,12], and Elholm *et al.* found an urban-rural gradient only for allergic asthma [16].

The centre-specific variations are in line with findings from the GABRIELA study showing the farm effect not to be universal as Polish farm children were less protected from asthma than German, Swiss and Austrian farm children [31]. In our study, the negative finding with regard to the urban-rural gradient may in part be due to the six categories of upbringing not being proportional to the microbial load and diversity. For instance, recent research suggests the microbial load from neighbouring farms to lower the risk of asthma among residents in rural areas not living on farms [6]. In addition, the categories may overlap and the subjects’ own definition of, for instance, a small town may vary between centres. We believe the measurement of upbringing reflects the urban-rural relationship in differing ways according to centre because of different population density, industrialization, lifestyle, distance between farms and city areas *etc.* This may in part explain the variations between centres, however any objective characteristics given for the different exposure categories would have allowed us a better understanding and interpretation of the patterns and the incremental differences when comparing two adjacent levels of urbanisation. In addition, behind the trend analyses lies a strong assumption that the difference in risk between two adjacent levels of urbanization is the same across the whole spectrum, and it may be questioned whether this is a reasonable assumption or not. Differences in consumption of unpasteurized milk may also contribute to centre variation, as it is suggested to be a part of the protective effect in many studies [12]. However, we believe that subjects growing up on livestock farms are more similar across the centres, and the variation in effect may be a result of differences in farming practice and size, as suggested by MacNeill *et al.* [31]. Apart from low power, we do not have any explanation for the outliers at the Danish farms without livestock.

The homogeneity of the five countries within the study population may influence the external validity. The Scandinavian countries (Denmark, Norway and Sweden) may be more similar to one another than to Estonia and Iceland. In addition, three of the seven centres are located in Sweden which means that Sweden accounts for 49% of the participants compared to, for instance, 9% from Estonia. This may contribute to an overrepresentation of Sweden, or the Scandinavian countries in general, and may skew the results. However, this skewing is met in the centre specific analyses.

## 5. Conclusions

In conclusion, this population-based study suggests an urban-rural gradient of asthma in a Northern European population, so that subjects growing up on a livestock farm had significantly less late-onset asthma than subjects growing up in cities. This finding supports the hypothesis that the microbial environment in early childhood may be of importance for subsequent development of asthma, as has been previously shown for sensitisation and allergy.

## Supplementary Files

Supplementary File 1
